# Dichlorido{*N*-[1-(2-pyrid­yl)ethyl­idene]ethane-1,2-diamine}copper(II)

**DOI:** 10.1107/S1600536809010149

**Published:** 2009-03-25

**Authors:** Qiang Wang, Cai-Feng Bi, Da-Qi Wang, Yu-Hua Fan

**Affiliations:** a College of Chemistry and Chemical Engineering, Ocean University of China, Shandong 266100, People’s Republic of China; b College of Chemistry and Chemical Engineering, Liaocheng University, Shandong 252059, People’s Republic of China

## Abstract

The title complex, [CuCl_2_(C_9_H_13_N_3_)], is mononuclear and contains a five-coordinate Cu^II^ atom. The geometry of the Cu^II^ atom can be described as tetra­gonal-pyramidal derived from the calculation of the value τ = 0.102. The three N atoms of the pyridine and ethane-1,2-diamine ligands and one Cl atom belong to the basal plane and the other Cl atom represents the axial position of the pyramid. The Cu atom is displaced by 0.2599 (2) Å from the basal plane towards the axial Cl atom. In the crystal, mol­ecules are linked into chains by inter­molecular N—H⋯Cl and C—H⋯Cl hydrogen bonds.

## Related literature

For general background, see: Coles *et al.* (1998 ▶). For the calculation of the geometry parameter τ in five-coordinate complexes, see: Addison *et al.* (1984 ▶).
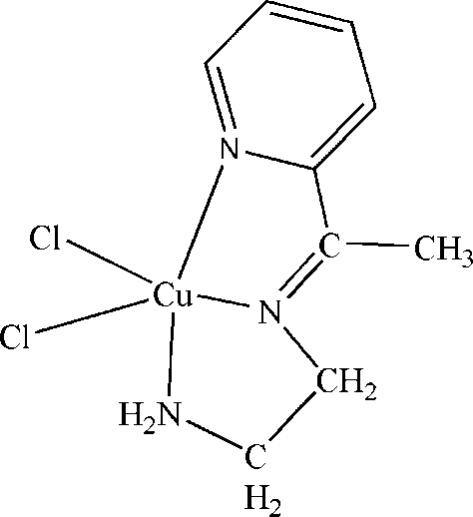


## Experimental

### 

#### Crystal data

[CuCl_2_(C_9_H_13_N_3_)]
*M* *_r_* = 297.66
Triclinic, 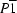

*a* = 7.2701 (10) Å
*b* = 8.8008 (12) Å
*c* = 9.5773 (15) Å
α = 82.940 (2)°
β = 76.289 (1)°
γ = 85.751 (2)°
*V* = 590.16 (15) Å^3^
*Z* = 2
Mo *K*α radiation
μ = 2.27 mm^−1^
*T* = 298 K
0.50 × 0.42 × 0.17 mm

#### Data collection

Siemens SMART CCD area-detector diffractometer
Absorption correction: multi-scan (*SADABS*; Sheldrick, 1996 ▶) *T*_min_ = 0.396, *T*_max_ = 0.699
2903 measured reflections
2021 independent reflections
1549 reflections with *I* > 2σ(*I*)
*R*_int_ = 0.034

#### Refinement

*R*[*F* ^2^ > 2σ(*F* ^2^)] = 0.053
*wR*(*F* ^2^) = 0.160
*S* = 1.04
2021 reflections
136 parameters
H-atom parameters constrained
Δρ_max_ = 0.91 e Å^−3^
Δρ_min_ = −0.81 e Å^−3^

### 

Data collection: *SMART* (Siemens, 1996 ▶); cell refinement: *SAINT* (Siemens, 1996 ▶); data reduction: *SAINT*; program(s) used to solve structure: *SHELXS97* (Sheldrick, 2008 ▶); program(s) used to refine structure: *SHELXL97* (Sheldrick, 2008 ▶); molecular graphics: *SHELXTL* (Sheldrick, 2008 ▶); software used to prepare material for publication: *SHELXTL*.

## Supplementary Material

Crystal structure: contains datablocks I, global. DOI: 10.1107/S1600536809010149/si2162sup1.cif

Structure factors: contains datablocks I. DOI: 10.1107/S1600536809010149/si2162Isup2.hkl

Additional supplementary materials:  crystallographic information; 3D view; checkCIF report

## Figures and Tables

**Table:** 

Cu1—N2	1.977 (5)
Cu1—N3	2.002 (5)
Cu1—N1	2.050 (5)
Cu1—Cl2	2.2659 (17)
Cu1—Cl1	2.4812 (16)

**Table:** 

N3—Cu1—N1	158.54 (19)
N2—Cu1—Cl2	164.66 (16)

**Table 2:** Hydrogen-bond geometry (Å, °)

*D*—H⋯*A*	*D*—H	H⋯*A*	*D*⋯*A*	*D*—H⋯*A*
N3—H3*A*⋯Cl1^i^	0.90	2.47	3.248 (5)	145
N3—H3*B*⋯Cl2^ii^	0.90	2.49	3.322 (5)	154
C6—H6⋯Cl2^iii^	0.93	2.77	3.666 (7)	161
C4—H4⋯Cl1^iv^	0.93	2.74	3.612 (7)	156
C1—H1*A*⋯Cl1^v^	0.96	2.80	3.726 (6)	161

Symmetry codes: (i) 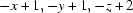; (ii) 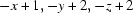; (iii) 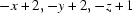; (iv) 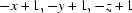; (v) 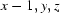.

**Fig. 1.**
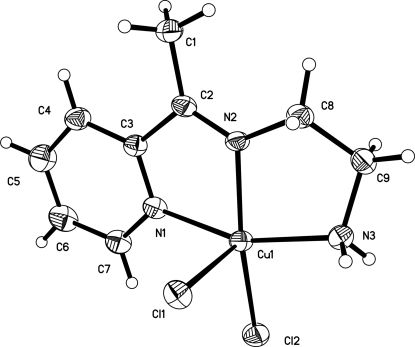
The structure of the title complex, showing 30% probability displacement ellipsoids and the atom-numbering scheme.

**Fig. 2.**
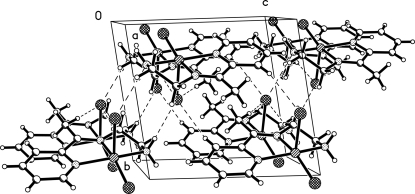
The crystal packing of the title complex, viewed approximately along the c axis.

## supplementary crystallographic information

### Comment

Schiff-base ligands have played an integral role in the development of coordination chemistry since the late 19t h century. The finding that metal complexes of these ligands are ubiquitous is a reflection of their facile synthesis, wide application and the accessibility of diverse structural modifications (Coles *et al.*, 1998). We report here the synthesis and crystal structure of the title complex, a new copper(II) complex, with a tridentate Schiff base ligand derived from the condensation of 2-acetylpyridine and diamine.

The molecular structure of the title complex is shown in Fig.1. The Cu^II^ atom is five-coordinated. The basal plane for a tetragonal-pyramidal geometry is defined by the atoms N1, N2, N3 and Cl2, their mean deviation from this plane is 0.025 Å, and the Cu atom juts out of this plane by 0.2599 (2) Å. The axial position of the pyramid is occupied by atom Cl1. For this point of view, a geometry parameter τ, which is defined τ = (β - α)/60, applicable to five-coordinate structures within the structural continuum between trigonal bipyramidal and tetragonal or rectangular pyramidal. For a perfect tetragonal symmetry τ is zero, and for a perfect trigonal-bipyramidal geometry τ becomes 1.0 (Addison *et al.* 1984). In the title compound, the largest angles within the four atoms N1, N2, N3, Cl2 are β = 164.66 (16)° for N2–Cu1–Cl2, and α = 158.54 (19)° for N1–Cu1–N3. Thus, τ is (164.66–158.54)/60 = 0.102, indicating a 90% rectangular pyramidal geometry. Selected geometric parameters are presented in Table 1. As seen in Fig. 2, the molecules are linked into chains by intermolecular N—H···Cl and C—H···Cl hydrogen bonds (Table 2).

### Experimental

2-acetylpyridine (10 mmol, 1205 mg) was added dropwise to a absolute ethanol (20 ml) of diamine (10 mmol, 611 mg). The mixture was heated under reflux with stirring for 3 h. An absolute ethanol solution (10 ml) of cupric chloride dihydrate (10 mmol, 1700 mg) was then added dropwise, and the mixture was stirred at room temperature for another 13 h. The solution was filtered off, the filterate was kept at room temperature for about three weeks, after which large green block-shaped crystals of the title complex suitable for X-ray diffraction analysis were obtained.

### Refinement

All H-atoms were positioned geometrically and refined using a riding model, with C—H = 0.90–0.97 Å, and N—H (amino) 0.90 Å, with *U*_iso_(H) =1.2*U*_eq_(C).

### Crystal data

**Table:**

[CuCl_2_(C_9_H_13_N_3_)]	*Z* = 2
*M**_r_* = 297.66	*F*(000) = 302
Triclinic, *P*1	*D*_x_ = 1.675 Mg m^−^^3^
Hall symbol: -P 1	Mo *K*α radiation, λ = 0.71073 Å
*a* = 7.2701 (10) Å	Cell parameters from 1739 reflections
*b* = 8.8008 (12) Å	θ = 2.3–27.9°
*c* = 9.5773 (15) Å	µ = 2.27 mm^−^^1^
α = 82.940 (2)°	*T* = 298 K
β = 76.289 (1)°	Block, green
γ = 85.751 (2)°	0.50 × 0.42 × 0.17 mm
*V* = 590.16 (15) Å^3^	

### Data collection

**Table:**

Siemens SMART CCD area-detector diffractometer	2021 independent reflections
Radiation source: fine-focus sealed tube	1549 reflections with *I* > 2σ(*I*)
graphite	*R*_int_ = 0.034
φ and ω scans	θ_max_ = 25.0°, θ_min_ = 2.2°
Absorption correction: multi-scan (*SADABS*; Sheldrick, 1996)	*h* = −5→8
*T*_min_ = 0.396, *T*_max_ = 0.699	*k* = −10→10
2903 measured reflections	*l* = −9→11

### Refinement

**Table:**

Refinement on *F*^2^	Primary atom site location: structure-invariant direct methods
Least-squares matrix: full	Secondary atom site location: difference Fourier map
*R*[*F*^2^ > 2σ(*F*^2^)] = 0.053	Hydrogen site location: inferred from neighbouring sites
*wR*(*F*^2^) = 0.160	H-atom parameters constrained
*S* = 1.04	*w* = 1/[σ^2^(*F*_o_^2^) + (0.0888*P*)^2^ + 0.8983*P*] where *P* = (*F*_o_^2^ + 2*F*_c_^2^)/3
2021 reflections	(Δ/σ)_max_ < 0.001
136 parameters	Δρ_max_ = 0.91 e Å^−^^3^
0 restraints	Δρ_min_ = −0.81 e Å^−^^3^

### Special details

**Table:**

Geometry. All e.s.d.'s (except the e.s.d. in the dihedral angle between two l.s. planes) are estimated using the full covariance matrix. The cell e.s.d.'s are taken into account individually in the estimation of e.s.d.'s in distances, angles and torsion angles; correlations between e.s.d.'s in cell parameters are only used when they are defined by crystal symmetry. An approximate (isotropic) treatment of cell e.s.d.'s is used for estimating e.s.d.'s involving l.s. planes.
Refinement. Refinement of *F*^2^ against ALL reflections. The weighted *R*-factor *wR* and goodness of fit *S* are based on *F*^2^, conventional *R*-factors *R* are based on *F*, with *F* set to zero for negative *F*^2^. The threshold expression of *F*^2^ > σ(*F*^2^) is used only for calculating *R*-factors(gt) *etc*. and is not relevant to the choice of reflections for refinement. *R*-factors based on *F*^2^ are statistically about twice as large as those based on *F*, and *R*- factors based on ALL data will be even larger.

### Fractional atomic coordinates and isotropic or equivalent isotropic displacement parameters (Å^2^)

**Table:**

	*x*	*y*	*z*	*U*_iso_*/*U*_eq_	
Cu1	0.53050 (9)	0.78810 (8)	0.77470 (7)	0.0327 (3)	
Cl1	0.7011 (2)	0.53529 (17)	0.80166 (16)	0.0397 (4)	
Cl2	0.7438 (2)	0.9433 (2)	0.81435 (18)	0.0476 (4)	
N1	0.6029 (6)	0.8157 (6)	0.5531 (5)	0.0341 (11)	
N2	0.3090 (6)	0.7056 (6)	0.7282 (5)	0.0347 (11)	
N3	0.3644 (7)	0.7751 (6)	0.9746 (5)	0.0357 (11)	
H3A	0.4022	0.6933	1.0288	0.043*	
H3B	0.3743	0.8597	1.0159	0.043*	
C1	0.1667 (9)	0.6172 (9)	0.5489 (7)	0.0527 (18)	
H1A	0.0658	0.5872	0.6302	0.079*	
H1B	0.2225	0.5283	0.5032	0.079*	
H1C	0.1171	0.6883	0.4809	0.079*	
C2	0.3123 (8)	0.6909 (7)	0.5987 (6)	0.0343 (13)	
C3	0.4844 (8)	0.7519 (7)	0.4929 (6)	0.0326 (13)	
C4	0.5180 (9)	0.7429 (8)	0.3465 (6)	0.0427 (15)	
H4	0.4342	0.6966	0.3070	0.051*	
C5	0.6828 (10)	0.8058 (9)	0.2596 (7)	0.0561 (19)	
H5	0.7108	0.8023	0.1601	0.067*	
C6	0.8026 (10)	0.8725 (9)	0.3213 (7)	0.0531 (18)	
H6	0.9125	0.9155	0.2646	0.064*	
C7	0.7586 (9)	0.8752 (7)	0.4685 (7)	0.0416 (15)	
H7	0.8408	0.9203	0.5105	0.050*	
C8	0.1613 (8)	0.6561 (8)	0.8557 (6)	0.0412 (15)	
H8A	0.1867	0.5504	0.8911	0.049*	
H8B	0.0381	0.6645	0.8321	0.049*	
C9	0.1656 (8)	0.7610 (8)	0.9685 (7)	0.0406 (14)	
H9A	0.1095	0.8611	0.9436	0.049*	
H9B	0.0931	0.7192	1.0622	0.049*	

### Atomic displacement parameters (Å^2^)

**Table:**

	*U*^11^	*U*^22^	*U*^33^	*U*^12^	*U*^13^	*U*^23^
Cu1	0.0302 (4)	0.0363 (5)	0.0324 (4)	−0.0047 (3)	−0.0096 (3)	0.0001 (3)
Cl1	0.0404 (8)	0.0339 (8)	0.0412 (8)	0.0017 (6)	−0.0079 (6)	0.0048 (6)
Cl2	0.0403 (9)	0.0487 (10)	0.0580 (10)	−0.0096 (7)	−0.0133 (7)	−0.0145 (8)
N1	0.031 (2)	0.035 (3)	0.033 (3)	−0.004 (2)	−0.007 (2)	0.007 (2)
N2	0.027 (2)	0.053 (3)	0.025 (2)	−0.009 (2)	−0.0086 (19)	0.002 (2)
N3	0.040 (3)	0.033 (3)	0.035 (3)	−0.001 (2)	−0.011 (2)	−0.001 (2)
C1	0.038 (3)	0.081 (5)	0.043 (4)	−0.016 (4)	−0.008 (3)	−0.015 (4)
C2	0.029 (3)	0.037 (3)	0.037 (3)	0.001 (2)	−0.012 (2)	0.001 (3)
C3	0.034 (3)	0.033 (3)	0.031 (3)	−0.002 (2)	−0.010 (2)	0.003 (2)
C4	0.042 (3)	0.051 (4)	0.036 (3)	−0.007 (3)	−0.013 (3)	0.001 (3)
C5	0.057 (4)	0.077 (5)	0.031 (3)	−0.009 (4)	−0.008 (3)	0.008 (3)
C6	0.044 (4)	0.060 (5)	0.047 (4)	−0.015 (3)	−0.003 (3)	0.015 (3)
C7	0.039 (3)	0.038 (3)	0.046 (4)	−0.009 (3)	−0.008 (3)	0.004 (3)
C8	0.033 (3)	0.053 (4)	0.037 (3)	−0.011 (3)	−0.007 (3)	0.001 (3)
C9	0.034 (3)	0.045 (4)	0.040 (3)	0.001 (3)	−0.006 (3)	−0.001 (3)

### Geometric parameters (Å, °)

**Table:**

Cu1—N2	1.977 (5)	C1—H1C	0.9600
Cu1—N3	2.002 (5)	C2—C3	1.498 (8)
Cu1—N1	2.050 (5)	C3—C4	1.376 (8)
Cu1—Cl2	2.2659 (17)	C4—C5	1.396 (9)
Cu1—Cl1	2.4812 (16)	C4—H4	0.9300
N1—C7	1.327 (7)	C5—C6	1.363 (10)
N1—C3	1.333 (7)	C5—H5	0.9300
N2—C2	1.257 (7)	C6—C7	1.372 (9)
N2—C8	1.467 (7)	C6—H6	0.9300
N3—C9	1.475 (7)	C7—H7	0.9300
N3—H3A	0.9000	C8—C9	1.512 (9)
N3—H3B	0.9000	C8—H8A	0.9700
C1—C2	1.479 (8)	C8—H8B	0.9700
C1—H1A	0.9600	C9—H9A	0.9700
C1—H1B	0.9600	C9—H9B	0.9700
			
N2—Cu1—N3	82.95 (19)	N2—C2—C3	114.1 (5)
N2—Cu1—N1	78.71 (18)	C1—C2—C3	120.6 (5)
N3—Cu1—N1	158.54 (19)	N1—C3—C4	122.8 (5)
N2—Cu1—Cl2	164.66 (16)	N1—C3—C2	114.0 (5)
N3—Cu1—Cl2	96.66 (15)	C4—C3—C2	123.2 (5)
N1—Cu1—Cl2	98.10 (14)	C3—C4—C5	117.5 (6)
N2—Cu1—Cl1	95.01 (16)	C3—C4—H4	121.3
N3—Cu1—Cl1	96.91 (15)	C5—C4—H4	121.3
N1—Cu1—Cl1	95.69 (14)	C6—C5—C4	119.6 (6)
Cl2—Cu1—Cl1	100.26 (6)	C6—C5—H5	120.2
C7—N1—C3	118.8 (5)	C4—C5—H5	120.2
C7—N1—Cu1	127.4 (4)	C5—C6—C7	119.0 (6)
C3—N1—Cu1	113.5 (4)	C5—C6—H6	120.5
C2—N2—C8	126.8 (5)	C7—C6—H6	120.5
C2—N2—Cu1	119.1 (4)	N1—C7—C6	122.4 (6)
C8—N2—Cu1	113.9 (4)	N1—C7—H7	118.8
C9—N3—Cu1	109.9 (4)	C6—C7—H7	118.8
C9—N3—H3A	109.7	N2—C8—C9	106.3 (5)
Cu1—N3—H3A	109.7	N2—C8—H8A	110.5
C9—N3—H3B	109.7	C9—C8—H8A	110.5
Cu1—N3—H3B	109.7	N2—C8—H8B	110.5
H3A—N3—H3B	108.2	C9—C8—H8B	110.5
C2—C1—H1A	109.5	H8A—C8—H8B	108.7
C2—C1—H1B	109.5	N3—C9—C8	108.7 (5)
H1A—C1—H1B	109.5	N3—C9—H9A	109.9
C2—C1—H1C	109.5	C8—C9—H9A	109.9
H1A—C1—H1C	109.5	N3—C9—H9B	109.9
H1B—C1—H1C	109.5	C8—C9—H9B	109.9
N2—C2—C1	125.3 (5)	H9A—C9—H9B	108.3

### Hydrogen-bond geometry (Å, °)

**Table:**

*D*—H···*A*	*D*—H	H···*A*	*D*···*A*	*D*—H···*A*
N3—H3A···Cl1^i^	0.90	2.47	3.248 (5)	145
N3—H3B···Cl2^ii^	0.90	2.49	3.322 (5)	154
C6—H6···Cl2^iii^	0.93	2.77	3.666 (7)	161
C4—H4···Cl1^iv^	0.93	2.74	3.612 (7)	156
C1—H1A···Cl1^v^	0.96	2.80	3.726 (6)	161

Symmetry codes: (i) −*x*+1, −*y*+1, −*z*+2; (ii) −*x*+1, −*y*+2, −*z*+2; (iii) −*x*+2, −*y*+2, −*z*+1; (iv) −*x*+1, −*y*+1, −*z*+1; (v) *x*−1, *y*, *z*.
